# Effect of Sarcopenia on the Increase in Liver Volume and Function After Portal Vein Embolization

**DOI:** 10.1007/s00270-024-03676-2

**Published:** 2024-02-28

**Authors:** Pieter J. W. Arntz, Pim B. Olthof, Remon Korenblik, Jan Heil, Geert Kazemier, Otto M. van Delden, Roelof J. Bennink, Steven W. M. Olde Damink, Ronald M. van Dam, Erik Schadde, Joris I. Erdmann

**Affiliations:** 1 Department of Surgery, Amsterdam UMC, University of Amsterdam, Amsterdam, The Netherlands; 2 Cancer Center Amsterdam, Amsterdam, The Netherlands; 3 Department of Surgery, Erasmus MC, Rotterdam, The Netherlands; 4 Department of Surgery, University Medical Center Groningen, Groningen, The Netherlands; 5 GROW School for Oncology and Developmental Biology, Maastricht University, Maastricht, The Netherlands; 6 Department of Surgery, Maastricht University Medical Center+, Maastricht, The Netherlands; 7 Institute of Physiology, University of Zurich, Zurich, Switzerland; 8 Department of General, Visceral and Transplant Surgery, University Hospital Frankfurt, Goethe-University Frankfurt, Frankfurt/Main, Germany; 9 Department of Surgery, Amsterdam UMC, Vrije Universiteit, Amsterdam, The Netherlands; 10 Department of Radiology and Nuclear Medicine, Amsterdam UMC, University of Amsterdam, Amsterdam, The Netherlands; 11 Department of Surgery, Rush University Chicago, Chicago, IL USA; 12 Department of General and Visceral Surgery, Cantonal Hospital Winterthur, Winterthur, Switzerland

**Keywords:** Sarcopenia, Portal vein embolization, Hepatobiliary scintigraphy, Liver function

## Abstract

**Purpose:**

Sarcopenia is associated with a decreased kinetic growth rate (KGR) of the future liver remnant (FLR) after portal vein embolization (PVE). However, little is known on the increase in FLR function (FLRF) after PVE. This study evaluated the effect of sarcopenia on the functional growth rate (FGR) after PVE measured with hepatobiliary scintigraphy (HBS).

**Methods:**

All patients who underwent PVE at the Amsterdam UMC between January 2005 and August 2017 were analyzed. Functional imaging by HBS was used to determine FGR. Liver volumetry was performed using multiphase contrast computed tomography (CT). Muscle area measurement to determine sarcopenia was taken at the third lumbar level (L3).

**Results:**

Out of the 95 included patients, 9 were excluded due to unavailable data. 70/86 (81%) patients were sarcopenic. In the multivariate logistic regression analysis, sarcopenia (*p* = 0.009) and FLR volume (FRLV) before PVE (*p* = 0.021) were the only factors correlated with KGR, while no correlation was found with FGR. 90-day mortality was similar across the sarcopenic and non-sarcopenic group (4/53 [8%] versus 1/11 [9%]; *p* = 1.000). The resection rates were also comparable (53/70 [75%] versus 11/16 [69%]; *p* = 0.542).

**Conclusion:**

FGR after PVE as measured by HBS appears to be preserved in sarcopenic patients. This is in contrast to KGR after PVE as measured by liver volumetry which is decreased in sarcopenic patients.

**Level of Evidence:**

Level 3b, cohort and case control studies.

**Supplementary Information:**

The online version contains supplementary material available at 10.1007/s00270-024-03676-2.

## Introduction

Cutoff values of future liver remnant (FLR) for safe liver resection, described in the literature, range from a minimum of 20–40%, depending on parenchymal quality and underlying liver disease [1–5]. To increase resectability and decrease risk of post-hepatectomy liver failure (PHLF), preoperative regenerative procedures such as portal vein embolization (PVE) before major liver resection are performed to induce contralateral hypertrophy of the FLR [6–10]. The degree of FLR hypertrophy after PVE varies and is dependent on patient, liver and procedure related factors [11]. In 20% of cases, patients fail to proceed for the planned resection [9], often due to inadequate growth and progression of disease.

Monitoring kinetic growth rate (KGR) to estimate liver growth after PVE provides valuable additional information in predicting PHLF [12]. Despite the recognition that KGR, as a time-dependent metric, provides better prediction of postoperative morbidity and mortality in small FLR compared to conventional volumetric measurement, both KGR and volumetric assessment at times either over- [13, 14] or underestimate [15] actual liver function after regenerative procedures.

Quantification of liver function based on technetium-99 m (^99m^Tc)-mebrofenin hepatobiliary scintigraphy (HBS) is a reliable method and may be used in addition to volumetry to select patients eligible for PVE. HBS is suggested to be more reliable than volumetry in the risk analysis of patients scheduled for major resection [16–19]. Performing HBS before and after PVE to measure the difference in liver function enables the derivation of functional growth rate (FGR) and potentially provides additional diagnostic value.

Sarcopenia is associated with adverse surgical outcomes and reduced resectability in patients scheduled for hepatectomy [20–24]. Moreover, sarcopenia is associated with an impaired KGR after PVE [23]. These findings raise the question whether FGR after PVE is preserved or impaired in sarcopenic patients.

In this study, we investigate whether sarcopenia influences FGR and KGR of the FLR following PVE in patients scheduled for major hepatectomy.

## Materials and Methods

### Study Design and Patients

This study was designed as a single-center retrospective cohort study. All patients who underwent PVE at the Amsterdam UMC between January 2005 and August 2017 were included. All data were obtained from a prospectively maintained database and were reported according to the STROBE guidelines [25].

### Liver Volumetry and Kinetic Growth Rate

Multiphase contrast computed tomography (CT) was carried out before and after PVE and primarily acquired for diagnostic purpose. The portal venous phase was used for the volumetric assessment. Total liver volume (TLV, cc) was determined by semiautomatic delineation of the liver in the pre- and postoperative CT scans in the axial plane using Syngo.via version 6.4 (Siemens Healthcare) and executed by the PVE performing interventional radiologist. Manual adjustments were made for incorrectly included extrahepatic structures. The sum of all delineated serial transverse CT slices defined TLV. Tumor margins were delineated to determine the tumor volume. The tumor volume was subtracted from TLV. Resection margins following the Couinaud classification of hepatic segments were applied to define the FLR volume (FLRV, cc). FLRV was divided by TLV to determine the volume share of the FLRV (FLRV share, %) and was expressed as % (FLRV% = FLRV × 100/TLV).

The difference in FLRV prior to and post-PVE defines ΔFLRV, also known as the degree of hypertrophy. KGR is generally defined as the difference in FLRV in mL before and after PVE, divided by the number of weeks between the two scan dates, representing the hypertrophic response per week. The KGR was expressed as %/week (KGR = ΔFLRV/TLV).

Standardized KGR (sKGR) is calculated in the same manner as KGR, but standardized TLV substituted for TLV in the calculation. Standardized TLV was calculated according to the formula: (18.51 × body weight [kg] + 191.8) [26].

### Liver Function Test and Functional Growth Rate

HBS is implemented in the routine workup of patients scheduled for major liver resection and in line with in house protocol and practical guidelines [27]. Quantitative assessment of liver function was performed by HBS as previously described [18, 19, 28]. A single-photon emission computed tomography (SPECT) acquisition in combination with a low-dose non-contrast enhanced CT was performed for anatomical mapping. Acquisition was done in closest proximity before PVE and three weeks after PVE, preferably on the same day as the multiphase contrast CT. ^99m^Tc-mebrofenin follows the hepatic bilirubin uptake and excretion pathway [29]. Segmentation was performed semiautomatically based on 20 consecutive SPECT images. An outline extraction model was applied to perform automatic delineation of the liver [27]. Resection margins to define the FLR are performed in the same manner as previously described. Post-processing of HBS scan data was performed with Hermes Medical Solutions software (version 2.13.0.22) and performed by one nuclear medicine physician with > 20 years of experience. Calculations were done according to the Ekman algorithm [30]. The calculation of FGR was performed in the same manner as for KGR.

### Portal Vein Embolization

An FLR function (FLRF) < 2.7%/min/m^2^ defined on the basis of HBS or an FLRV < 30% was indicative for PVE of the right or left portal system [18, 19]. An additional cutoff value for FLRF in perihilar cholangiocarcinoma was set at 8.5%/min based on the results of a study conducted in 2017 [31]. Specific to the patient, either an ipsi- or contralateral PVE approach was performed. Embolization of the portal vein branches was done using polyvinyl alcohol particles (300–500 nm) and coils. Embolization was performed by four interventional radiologists with > 5 years of experience.

### Sarcopenia

The HBS supplementary CT was used for skeletal muscle area (SMA) measurement in the axial plane of the mid-third lumbar vertebra (L3), using Osirix Lite v12.0.2 (Pixmeo SARL, Switzerland) imaging software. Alternative CT scans were used in missing cases, provided that the scans were acquired within one month timespan of the HBS acquisition date. The threshold range was set from − 30 to + 150 Hounsfield units for the delineation of the SMA, which was standardized to height (m^2^) to define the skeletal muscle index (SMI). Sarcopenia cutoff values were defined by SMI, body mass index (BMI) and gender. For females, SMI < 41 cm^2^/m^2^ was considered indicative of sarcopenia, regardless of BMI. For males, SMI < 43 cm^2^/m^2^ with a BMI < 25 and SMI < 53 cm^2^/m^2^ with a BMI > 25 was considered indicative of sarcopenia [20]. In the literature reported, ‘most optimal’ cutoff values for sarcopenia were used [32, 33].

### Statistics

All categorical variables were shown as numbers with percentages. Differences were tested using Chi-square or Fisher’s exact tests. All continuous variables were shown as medians with interquartile range (IQR), and differences were tested using Mann–Whitney U-tests. Multivariate analysis on factors that were associated with KGR or FGR was performed using binary logistic regression. Patients were categorized based on a KGR or FGR above or below the median value. All statistical analyses were performed using SPSS (version 26, IBM, Chicago, IL), and all graphs were generated using GraphPad Prism (version 9, GraphPad Inc., La Jolla, CA).

## Results

A total of 95 patients underwent preoperative PVE during the study period. For 9 patients, CT imaging at L3 level for sarcopenia measurement was not available and these patients were excluded from the analyses. Out of the 86 patients, 70 (81%) were sarcopenic. Baseline and disease characteristics are shown in Table 1.

**Table 1:** Baseline characteristics of participants

Variates	Sarcopenia (no) (*n* = 16)	Sarcopenia (yes) (*n* = 70)	*p* value
Age*, median (IQR)*	63 (56–70)	64 (59–70)	0.701
Male sex*, n (%)*	11 (69)	46 (66)	1.000
Weight*, kg, median (IQR)*	74 (67–80)	77 (66–86)	0.617
Height*, cm, median (IQR)*	177 (168–180)	176 (170–182)	0.571
BMI*, kg/m*^*2*^*, median (IQR)*	24.5 (23.3–25.3)	24.6 (22.1–27.7)	0.731
BSA*, m*^*2*^*, median (IQR)*	1.9 (1.8–2.0)	1.9 (1.8–2.1)	0.610
Diabetes*, n (%)*	-	6 (9)	0.589
Tumor type*, n (%)*			0.646
*CRLM*	7 (44)	43 (61)	
*HCC*	2 (13)	4 (6)	
*PHC*	6 (38)	16 (23)	
*IHC*	-	2 (3)	
*Other*	1 (6)	5 (7)	
Preoperative chemotherapy*, n (%)*	8 (50)	29 (41)	0.584
Cirrhosis*, n (%)*	2 (6)	1 (3)	0.465
SMA*, cm*^*2*^*, median (IQR)*	140 (135–148)	122 (102–141)	**0.002**
SMI* cm*^*2*^*/m*^*2*^*, median (IQR)*	46 (44–49)	40 (36–43)	**< 0.001**
TLV pre-PVE*, mL, median (IQR)*	1880 (1547–2527)	1720 (1443–2073)	0.155
FLRV pre-PVE*, mL, median (IQR)*	472 (377–706)	492 (349–632)	0.412
FLRV pre-PVE*, %, median (IQR)*	31 (25–38)	30 (23–34)	0.329
TLV post-PVE*, mL, median (IQR)*	1932 (1621–2407)	1687 (1428–1964)	0.070
FLRV post-PVE*, mL, median (IQR)*	760 (617–980)	638 (499–794)	**0.025**
FLRV post-PVE*, %, median (IQR)*	44 (41–57)	40 (34–45)	**0.005**
Days between PVE and CT scan*, median (IQR)*	21 (21–28)	22 (21–25)	0.504
KGR,* %/week, median (IQR)*	4.8 (3.3–6.4)	3.0 (2.2–4.0)	**0.002**
FLRV increase*, %, median (IQR)*	52 (21–68)	34 (24–55)	0.272
Total liver function pre-PVE*, %/min, median (IQR)*	13.8 (11.6–15.2)	15.7 (12.1–14.5)	0.094
FLRF pre-PVE*, %/min, median (IQR)*	4.6 (4.2–5.7)	4.4 (3.3–5.8)	0.323
FLRF pre-PVE, *%/min/m*^*2*^*, median (IQR)*	2.4 (2.3–3.0)	2.2 (1.8–3.0)	0.248
Total liver function post-PVE*, %/min, median (IQR)*	13.6 (11.0–16.2)	14.4 (11.8–15.9)	0.773
FLRF post-PVE*, %/min, median (IQR)*	8.3 (6.9–9.5)	7.5 (5.3–9.0)	0.202
FLRF post-PVE, *%/min/m*^*2*^*, median (IQR)*	4.2 (3.5–5.0)	3.7 (2.6–4.7)	0.253
Days PVE and HBS scan*, median (IQR)*	21 (20–23)	22 (21–23)	0.347
FGR*, %/week, median (IQR)*	0.90 (0.19–1.49)	0.88 (0.44–1.26)	0.938
FLRF increase*, %, median (IQR)*	60 (9–99)	53 (36–89)	0.903
Resection rate*, n (%)*	11 (69)	53 (75)	0.542

*BMI* body mass index, *BSA* body surface area, *CRLM* colorectal Liver metastases, *HCC* hepatocellular carcinoma, *PHC* perihilar cholangiocarcinoma, *IHC* intrahepatic cholangiocarcinoma, *SMA* skeletal muscle area, *SMI* skeletal muscle index, *TLV* total liver volume, *FLR(V)(F)* future liver remnant (volume)(function), *PVE* portal vein embolization, *CT* computed tomography, *KGR* kinetic growth rate, *FGR* functional growth rate, *IQR* interquartile ranges

Values are presented as median and interquartile range (range) for continuous variables and as number (percentages) for categorical variables. *p* < 0.05 is defined as significant difference and highlighted in bold style

Baseline and disease characteristics were similar between sarcopenic and non-sarcopenic patients. While all volume and function parameters were similar at baseline before PVE when comparing the sarcopenic and the non-sarcopenic patients, only KGR and FLRV were significantly higher in the non-sarcopenic patients after PVE, when measured in mL or calculated as percentage of TLV in case of FLRV and increase relative to the FLRV before PVE in case of KGR. Liver function parameters were all similar between the two groups.

Segment IV was embolized in five patients, all of whom were males. Four of these patients were sarcopenic, of which two had a BMI > 25. Three had the diagnosis of perihilar cholangiocarcinoma, and two had the diagnosis of colorectal liver metastases.

The resection rate was similar in both sarcopenic and non-sarcopenic patients (53/70 [75%] versus 11/16 [69%]; *p* = 0.542). Five non-sarcopenic patients were not resected, due to local tumor progression (*n* = 3), peritoneal metastases (*n* = 1) and insufficient hypertrophy of the FLR (*n* = 1). Out of the 17 sarcopenic patients that were not resected, reasons comprised tumor progression (*n* = 12), technically unresectable (*n* = 1), radiofrequency ablation only due to a preferred change in treatment planning (*n* = 1) and liver involvement at exploration (*n* = 1). The remaining two patients had insufficient FLR hypertrophy.

Among the resected patients, 90-day mortality was similar across the sarcopenic and non-sarcopenic group (4/53 [8%] versus 1/11 [9%]; *p* = 1.000). The percentage of patients with PHLF according to the International Study Group of Liver Surgery criteria were identical to the 90-day morality rates and therefore all mortality was liver failure related. Uni- and multivariable analysis to identify factors that are associated with the KGR are shown in Table 2. Besides the baseline FLR in percentage, sarcopenia was associated with a poor KGR at multivariable analysis. Sarcopenia was not associated with the FGR.

**Table 2:** Uni- and multivariable analysis for kinetic growth rate and functional growth rate (FGR)

	Univariable analysis KGR		Multivariable analysis KGR	
	Odds ratio (95% CI)	*p* value	Odds ratio (95% CI)	*p* value
Age	0.99 (0.95–1.02)	0.985	0.99 (0.95–1.03)	0.647
Female sex	1.37 (0.55–3.42)	0.497	2.10 (0.73–6.07)	0.172
Chemotherapy	0.81 (0.34–1.92)	0.634	0.48 (0.17–1.40)	0.180
Diabetes	0.76 (0.14–3.98)	0.756	1.45 (0.22–9.45)	0.699
Cirrhosis	0.37 (0.03–4.18)	0.365	0.17 (0.01–2.86)	0.219
FLRV pre-PVE	0.95 (0.90–1.01)	0.088	**0.93 (0.87–0.99)**	**0.021**
Sarcopenia	**0.24 (0.06–0.93)**	**0.039**	**0.13 (0.03–0.60)**	**0.009**

	Univariable analysis FGR		Multivariable analysis FGR	
	Odds ratio (95% CI)	*p* value	Odds ratio (95% CI)	*p* value
Age	0.99 (0.95–1.03)	0.597	0.99 (0.95–1.03)	0.566
Female sex	0.78 (0.32–1.91)	0.586	0.69 (0.37–1.77)	0.435
Chemotherapy	1.30 (0.55–3.08)	0.554	1.29 (0.50–3.32)	0.604
Diabetes	0.38 (0.07–2.18)	0.277	0.42 (0.06–2.83)	0.374
Cirrhosis	1.61 (0.14–18.44)	0.702	1.90 (0.15–23.57)	0.619
FLRF pre-PVE	0.94 (0.73–1.21)	0.635	0.95 (0.73–1.24)	0.702
Sarcopenia	0.98 (0.33–2.92)	0.969	1.05 (0.34–3.27)	0.929

*KGR* kinetic growth rate, *FGR* functional growth rate, *FLR(V)(F)* future liver remnant (volume)(function), *PVE* portal vein embolization

Values are presented as odds ratio and 95% confidence interval (range). *p* < 0.05 is defined as significant and highlighted in bold style

## Discussion

This study reported on the influence of sarcopenia on FGR after PVE measured by HBS. The data in the current study do not support that FGR is negatively affected by sarcopenia while KGR is negatively affected by sarcopenia, as reported in prior studies [23, 34, 35]. However, no difference in resection rate was found between sarcopenic and non-sarcopenic patients. Morbidity and mortality rates were similar in both groups.

Alternative methods to quantify liver function comprise liver maximum capacity test (LiMAx), indocyanine green (ICG) retention test and several hepatobiliary phase magnetic resonance indices [36–38]. Liver function, as measured by LiMAx, was not affected by sarcopenia [39]. Both LiMAx, which evaluates metabolic activity, and HBS, which evaluates hepatic uptake and excretion in the biliary system, indirectly substitute for liver function. Both only measure a single liver function and might assess certain aspects of liver function that are not affected by sarcopenia. In a previous study, sarcopenia was associated with higher ICG retention values at 15 min (*p* = 0.049) indicating liver dysfunction [21]. However, sarcopenia cutoff values validated in patients with colorectal liver metastases were used in a cohort with only hepatocellular carcinoma, doubting the validity.

Sarcopenic patients experience an increased rate of major postoperative complications (Clavien–Dindo classification ≥ IIIa) after hepatectomy, are more susceptible to infections and intra-abdominal abscess, and generally have an extended hospital stay compared to non-sarcopenic patients [40, 41]. Sarcopenia might be indirectly associated with PHLF due to increased susceptibility to other complications, such as those that can lead to secondary liver failure. Therefore, adverse surgical outcome related to sarcopenia may not be represented by HBS measurement, as it predominantly predicts PHLF with a fulminant and rapid onset related to an insufficient FLR [42].

Despite efforts to reduce PHLF through preoperative assessment of liver function, liver failure and related mortality remain a frequent complication after resection of perihilar cholangiocarcinoma [42]. A more liberal approach to perform preoperative PVE was instated to decrease liver failure and mortality rates [43, 44]. The strategy regarding PVE explains the relatively high preoperative FLRV in the current cohort.

No difference in morbidity and mortality was observed between sarcopenic and non-sarcopenic patients in the current study cohort that underwent PVE, while previous studies report that sarcopenia negatively affects overall outcome after liver and other types of surgery [24, 45–49]. This seeming contradiction may be explained by the fact that all patients in this group underwent PVE, which is found to reduce the risk of PHLF and mortality in high-risk resections leading to a low rate of comparable events [50]. A recent study described similar rates of morbidity and mortality, which was linked to the protective traits of PVE [23].

This study has several limitations. First of all, the relatively low sample size 86 patients included in the study might obscure the effect of sarcopenia on FGR in liver function following PVE, potentially leading to a type II error. In addition, the small sample size of the non-sarcopenic group also increases the risk of type II error and may affect the validity of the results. However, differences in FGR were small between groups. The selection criteria used to assess eligibility for PVE and subsequent surgery also introduces selection bias. Although a randomized trial would decrease selection bias, denying patients a generally accepted procedure that improves surgical outcome such as PVE is ethically unacceptable. Not all patients in this study ultimately underwent planned resection and histological scoring of liver parenchymal quality characteristics. Full blood work and therefore laboratory results were not available in all patients as well. Nevertheless, a strong point of the study design is that it comprises a unique single-center cohort, reflecting uniform management of the patients. As a result of the relatively dated and long inclusion period, risk of era bias is introduced. Lastly, embolization in this study was performed with polyvinyl alcohol particles and coils instead of N-butyl cyanoacrylate glue, which is the current standard. The latter induces more hypertrophy, which could potentially mitigate differences between groups. Nevertheless, differences in KGR between the sarcopenic and non-sarcopenic groups were still significant.

In conclusion, FLRF as measured by HBS and FGR following PVE appear to be preserved in sarcopenic patients. This is in contrast to the negative influence of sarcopenia on liver volume and KGR observed after PVE and points to a preservation of function over volume in sarcopenic liver regeneration that requires further investigation.

### Supplementary Information

Below is the link to the electronic supplementary material.

Supplementary file1 (DOCX 353 kb)
