# Supplementary material for: Effect of Sarcopenia on the Increase in Liver Volume and Function After Portal Vein Embolization
Source: Cardiovasc Intervent Radiol. 2024 Feb 28;47(5):642–9. doi: 10.1007/s00270-024-03676-2 (PMC11074030; doi:10.1007/s00270-024-03676-2)

**Effect of Sarcopenia on the Increase in Liver Volume and Function After Portal Vein Embolization**

**CardioVascular and Interventional Radiology**

*Pieter JW Arntz^1,2^, Pim B Olthof^3,4^ MD PhD, Remon Korenblik^5,6^ MD, Jan Heil^7,8^ MD, Geert Kazemier^2,9^ MD PhD, Otto M van Delden^10^ MD PhD, Roelof J Bennink^10^ MD PhD, Steven WM Olde Damink^5,6^ MD PhD, Ronald M van Dam^4,5^ MD PhD, Erik Schadde^7,11,12^ MD PhD, +Joris I Erdmann^1,2^ MD PhD

*1. Department of Surgery, Amsterdam UMC, University of Amsterdam, The Netherlands*

*2. Cancer Center Amsterdam, The Netherlands*

*3. Department of Surgery, Erasmus MC, Rotterdam, The Netherlands*

*4.. Department of Surgery, University Medical Center Groningen, Groningen, the Netherlands*

*5. GROW School for Oncology and Developmental Biology, Maastricht University, The Netherlands*

*6. Department of Surgery, Maastricht University Medical Center+, The Netherlands*

*7. Institute of Physiology, University of Zurich, Zurich, Switzerland*

*8. Department of General, Visceral and Transplant Surgery, University Hospital Frankfurt, Goethe-University Frankfurt, Frankfurt/Main, Germany*

*9. Department of Surgery, Amsterdam UMC, Vrije Universiteit, Amsterdam, the Netherlands*

*10. Department of Radiology and Nuclear Medicine, Amsterdam UMC, University of Amsterdam, The Netherlands*

*11. Department of Surgery, Rush University Chicago, Illinois, USA*

*12. Department of General and Visceral Surgery, Cantonal Hospital Winterthur, Winterthur, Switzerland*

^*^ First author

^+^ Senior author

**Correspondence:** Joris Erdmann, MD PHD FEBS

Department of Surgery

Amsterdam UMC, Location VU | Location AMC

De Boelelaan 1117 | Meibergdreef 9

1081HV | 1105AZ

Phone: +31 6 24 63 69 68

Email: [j.i.erdmann@amsterdamumc.nl](mailto:j.i.erdmann@amsterdamumc.nl)

**Figure 1**: Scatter plot describing the distribution of kinetic growth rate (KGR) (A) and functional growth rate (FGR) (B) in sarcopenic and non-sarcopenic patients


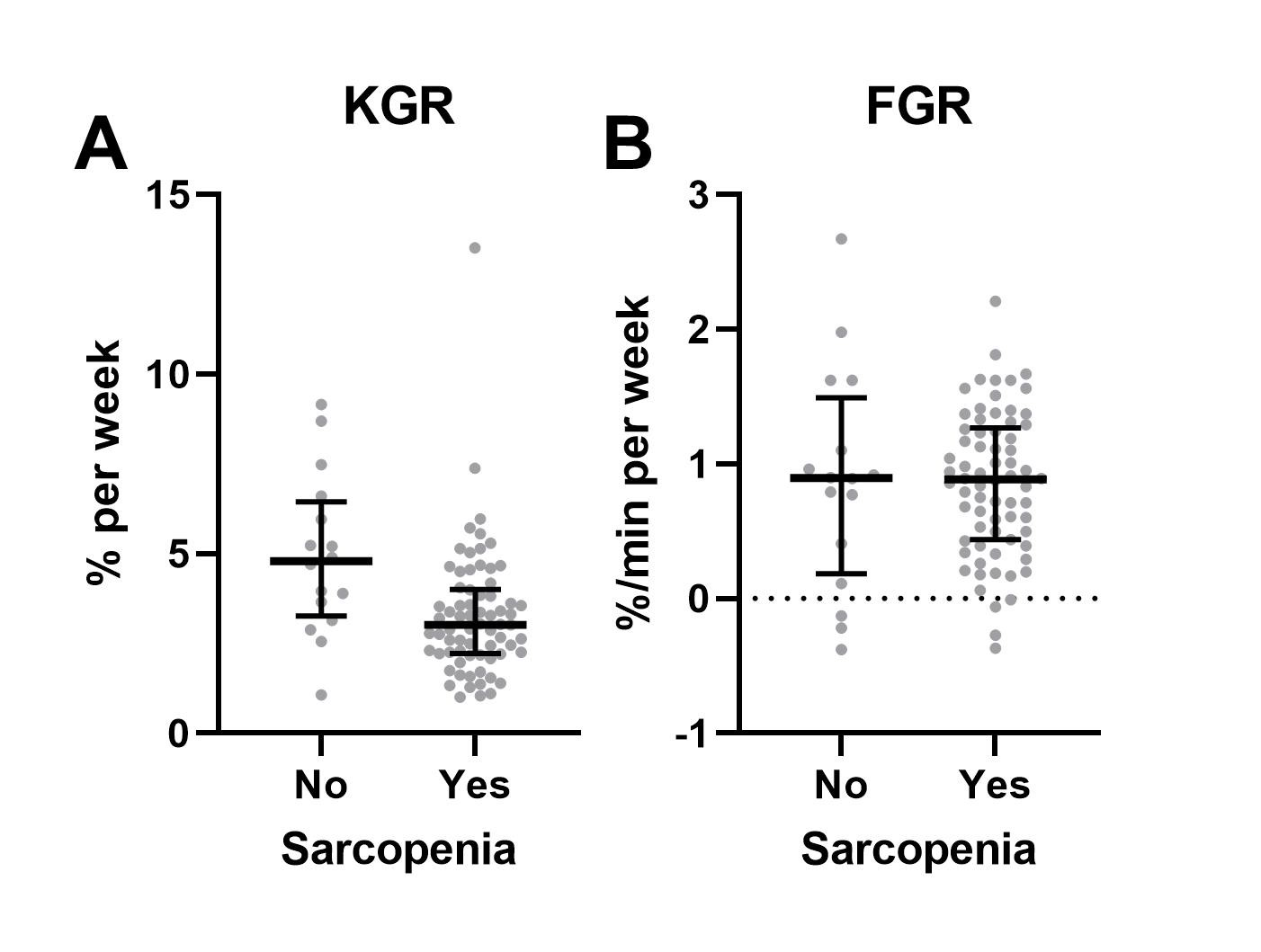


**Figure 2**: Scatter plot describing the correlation between kinetic growth rate (KGR) and functional growth rate (FGR) (A), volumetric increase and functional increase (B), and standardized (s)KGR and KGR (C) in sarcopenic and non-sarcopenic patients


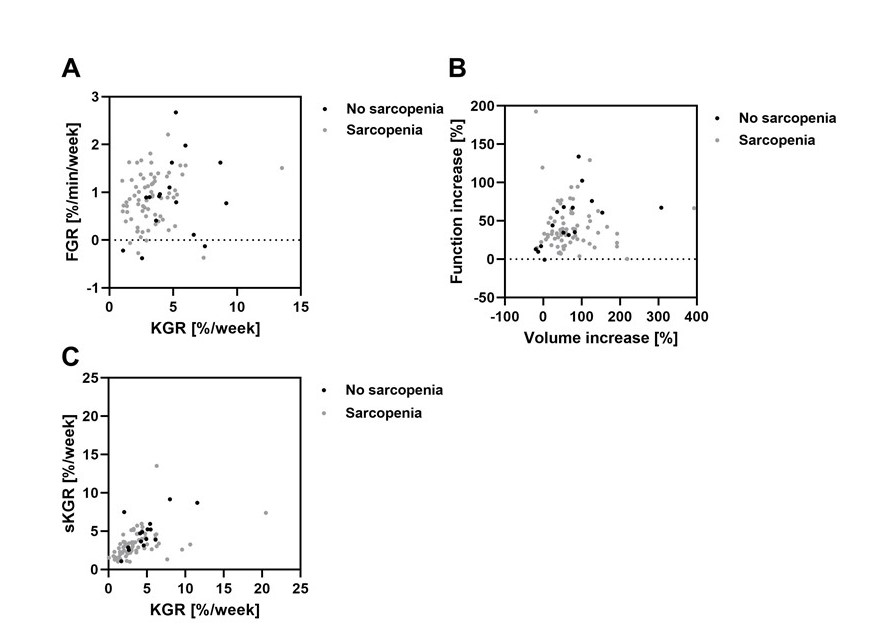

Supplement: Supplementary file 1 — Supplementary file1 (DOCX 353 kb) [file 270_2024_3676_MOESM1_ESM.docx]
